# Error in Byline and Results

**DOI:** 10.1001/jamanetworkopen.2024.53497

**Published:** 2024-12-02

In the Consensus Statement titled “Guidelines for the Prevention, Diagnosis, and Management of Urinary Tract Infections in Pediatrics and Adults: A WikiGuidelines Group Consensus Statement,”^1^ published November 4, 2024, there were errors in the byline and the Results section. There was a typo in Dr Moore’s name. The reference to Table 5 in the Results section has been removed, and questions 36 and 37 have been placed in numerical order. This article has been corrected.^1^
